# High-stage Device-related Pressure Injury Reduction in a Neonatal Intensive Care Unit: A Quality Improvement Project

**DOI:** 10.1097/pq9.0000000000000554

**Published:** 2022-06-14

**Authors:** Laurel B. Moyer, Denise L. Lauderbaugh, Katherine Worten, Chelsea Carter, Peggy Holub, Rose A. Santos Manrique, Judy H. Bergman, Mary Anne Dilloway, Marisha Hamid, Linda Glenn

**Affiliations:** * Division of Neonatology, Department of Pediatrics, University of California San Diego, La Jolla, Calif.; † Rady Children’s Hospital San Diego, San Diego, Calif.; ‡ National Children’s Hospital, Washington, N.C.

## Abstract

**Introduction:**

Pressure injuries are a common complication in neonatal intensive care settings, and neonates are at high risk for this hospital-acquired condition. Pressure injury rates in the neonatal intensive care unit (NICU) at Rady Children’s Hospital were higher than reported national comparisons in 2018. Device-related high-stage hospital-acquired pressure injuries (HAPI) were the most common injury source. We aimed to reduce the rate of device-related high-stage HAPIs per 1,000 patient days by 30% within 12 months.

**Methods:**

We formed an interdisciplinary quality improvement (QI) task force to address device-related injury. The team identified opportunities and interventions and created care bundles using QI methodology. To engage staff, device-related HAPI data were shared at nursing and respiratory therapy meetings. The team and stakeholders chose metrics. Outcome, process, and balancing measures were analyzed and displayed on statistical process control charts.

**Results:**

Device-related HAPIs were reduced by 60% from 0.94 to 0.37 per 1,000 patient days. electroencephalography and CPAP-related events were decreased to 0 and sustained for 10 months.

**Conclusions:**

Interprofessional collaboration, and a strong reliance on data were keys to reducing high-stage pressure injuries. This approach can be replicated and implemented by other units experiencing similar challenges.

## INTRODUCTION

Hospital-acquired pressure injuries (HAPI) among children in the intensive care setting leads to significant morbidity and associated costs due to prolonged illness, immobilization, and increased device utilization.^1–11^ Neonates are especially at high risk of HAPI secondary to their prolonged need for respiratory device use, low subcutaneous fat stores, and premature skin.^5,6,8–10^

The National Pressure Injury Advisory Panel classifies pressure injury using a staging system to ensure uniformity in reporting.^12^ The National Pressure Injury Advisory Panel defines stage 1 injury as intact skin with localized nonblanchable erythema; stage 2 is partial thickness loss with dermis exposed; stage 3 is full-thickness loss; stage 4 is full-thickness skin and tissue loss; and unstageable is full-thickness skin and tissue loss where tissue damage cannot be confirmed because it is covered by eschar.^12^ The Solutions for Patient Safety (SPS) Network is a collaborative with over 145 pediatric hospitals working together to decrease serious harm from high-stage HAPI, defined as stage 3, 4, and unstageable HAPI.^2,3,13,14^

**Fig. 1.**
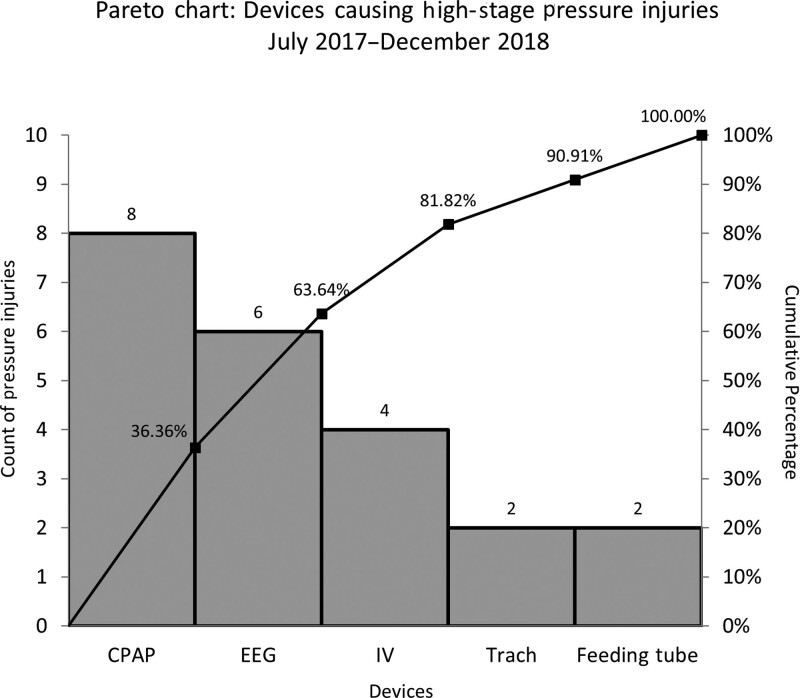
Pareto chart of device-related HAPI events.

A review of high-stage HAPI rates at Rady Children’s Hospital Level IV neonatal intensive care unit (NICU) revealed higher pressure injury rates compared to the other SPS network hospitals, prompting a detailed review of the most common causes for high-stage HAPI in the NICU. In the review, device-related high-stage HAPI accounted for 84% of all pressure injuries. Of these device-related injuries, respiratory device-related injury, specifically those resulting from occlusive continuous positive airway pressure (CPAP) devices, was the leading contributor followed by those related to electroencephalography (EEG) electrodes (Fig. 1).

Previous studies demonstrate the value of preventive care bundles to reduce pressure injury.^2,3,7,9,15^ Implementation of preventive care bundles in 99 pediatric hospitals resulted in a 57% reduction in pressure injuries.^15^ Several authors have shown that an interdisciplinary team is more effective at reducing pressure injuries than a single discipline.^1, 3, 5–7, 16, 17^ SPS participating hospitals had varied participation with the implementation of nursing interventions,^15^ which represented an opportunity for our team to fully implement preventive bundles and bedside care interventions. The aim of this improvement effort was to reduce the rate of device-related high-stage HAPIs per 1,000 patient days by 30% within 12 months. We outline our success engaging interprofessional collaboration to develop key drivers and decrease the rate of high-stage HAPI, including device-related HAPI.

## METHODS

### Setting

Rady Children’s Hospital is an academic, nonprofit, freestanding children’s hospital located in San Diego, Calif. The 64 bed, Level IV NICU has over 800 admissions annually and a heterogeneous population ranging from 23-week premature infants, to 6-month-old medically fragile infants, and those admitted from home with respiratory failure. Over 100 patients are admitted annually with a primary diagnosis of neurologic concern, including, but not limited to hypoxic-ischemic encephalopathy. More than 150 patients are admitted annually with the primary diagnosis of respiratory concern. The NICU staff includes an interprofessional team of neonatologists, neonatology fellows, advanced practice nurses, registered nurses (RNs), respiratory therapists (RTs), dedicated clinical pharmacists, dieticians, and social workers.

## PLANNING THE INTERVENTIONS

We formed an interprofessional QI task force consisting of nurses, RTs, physicians, wound care specialists and EEG technicians in January 2019 to address the problem of high-stage HAPI related to EEG leads. The team identified two major causes with differing processes for improvement of device-related high-stage HAPI. We created two working groups (CPAP and EEG) to simultaneously address these areas.

**Fig. 2.**
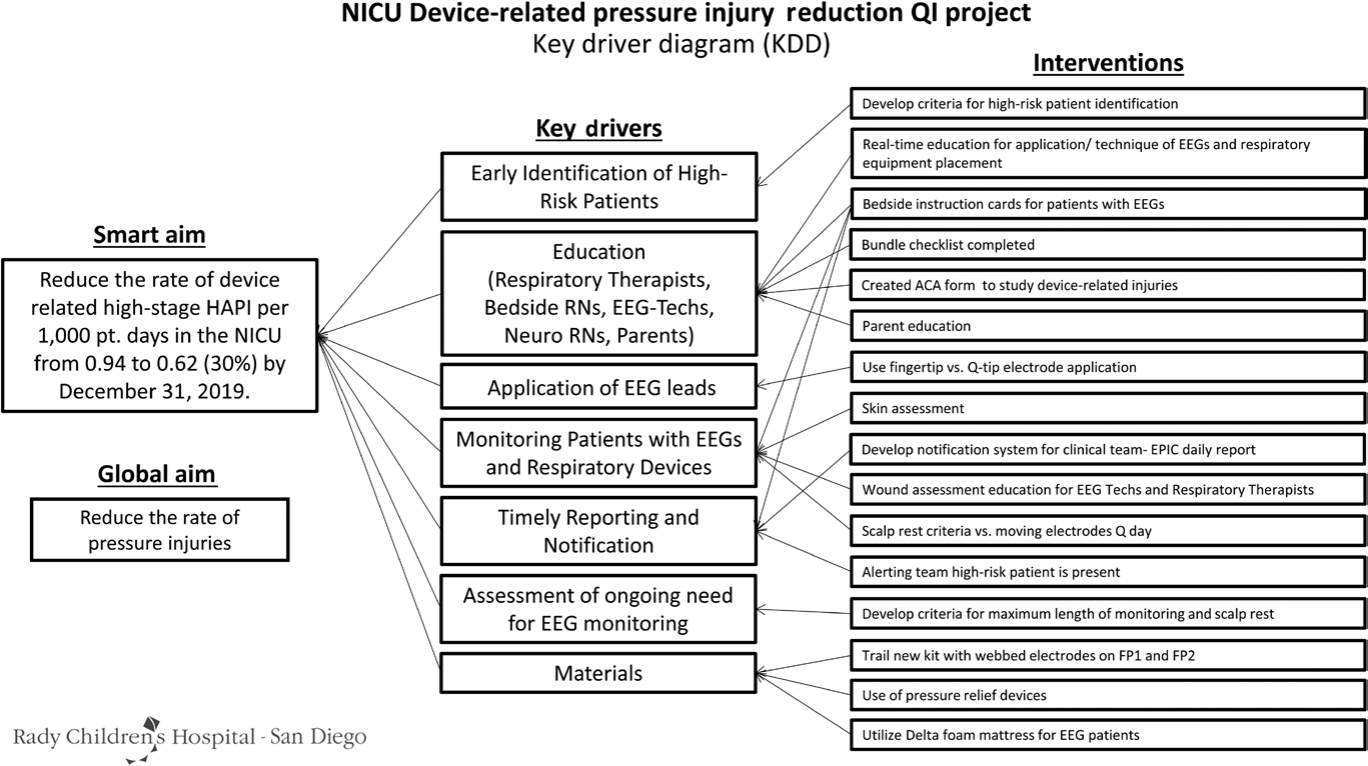
Key driver diagram for device-related HAPI events in the NICU.

Using the Model for Improvement,^18^ the EEG device-related HAPI team developed a fishbone diagram to illustrate and organize information regarding obstacles and variation in the care processes.^18^ The fishbone diagram causal groupings informed the development of a key driver diagram (Fig. 2). After analyzing the data, identifying barriers, and classifying key drivers, we created an EEG monitoring bundle for any patient requiring continuous EEG monitoring including a detailed injury review report.

Based on feedback from frontline staff, the respiratory device QI team recognized that there were educational gaps and a lack of standardization in processes. This prompted an initial focus on educational updates, followed by creating a PI prevention care bundle, including a plan to review all high-stage pressure injuries.

## INTERVENTION

The EEG device-related HAPI team developed an EEG monitoring bundle through several PDSA cycles. This bundle included a specialized (1) EEG electrode kit; (2) placement of a transparent contact dressing under each electrode; (3) a pressure-relieving foam mattress overlay; and (d) a bedside instruction care card. An EEG application checklist was created to guide EEG technicians and RNs through the bundle elements. Simultaneously, to promote the development of additional PDSAs, an apparent cause analysis (ACA) form was completed when an injury occurred. Each event was reviewed at a monthly quality improvement meeting to determine if the event was preventable with current processes or if changes were needed. Through an iterative process, we revised the EEG guideline to include daily skin checks and earlier EEG electrode removal to allow for scalp rest.

The respiratory device-related QI team initially focused on educational updates regarding placement of CPAP and skin protection for the RTs and nurses. Once education was complete, the team developed a prevention bundle modeling standard elements from the SPS skin assessment module, medical device rotation/reposition, patient positioning, appropriate surface, and moisture management.^14^ Elements of the bundle were trialed, and we reviewed feedback from bedside staff regarding which were most feasible and effective. The bundle consisted of (1) foam dressing use for any respiratory devices; (2) proper device fit assessment; and (3) rotation of CPAP/BIPAP masks/prongs every 4 hours (exception extremely low birthweight [ELBW] patients every 6 hours with touch times), and d) an assessment of all pressure points by two licensed care providers. A newly created skin integrity guide served as a checklist based on the type of respiratory device, device securing product, specifics for device use, assessment and documentation standards, and education regarding when to use a skin barrier appropriately. Respiratory therapy skin champions performed audits and gave feedback to the bedside providers regarding improvement opportunities. An ACA form specific to respiratory devices was created to identify additional gaps and areas for improvement. We shared respiratory device-related HAPI and bundle compliance data at nursing and respiratory therapy meetings to promote transparency, collaboration, and real-time problem-solving.

Several interventions were adopted by both teams to standardize practice. The Braden QD scale was utilized to identify and prioritize each patient’s level of risk for injury in both projects.^9,19,20^ Patients with a Braden QD score ≥ 13 required Active Skin Surveillance to proactively identify and treat potential injuries. This process was tested on a small scale in the cardiac intensive care unit (ICU), then within the NICU. It was adopted as an NICU guideline in December 2019 and subsequently implemented in all ICUs. During active surveillance, a skin assessment was performed, and audits completed comprising the following components: Braden QD scoring accuracy, appropriate padding under all medical devices, device rotation, patient positioning, use of appropriate mattress overlay, and moisture management. We defined active surveillance as the periodic (at least monthly) head-to-toe assessment of every patient in the NICU by a team including at least a Wound Treatment Associate (WTA) and bedside nurse.^3^ WTAs and quality management (QM) nursing partners completed these audits. The WTA Program developed by the Wound, Ostomy and Continence Nurses (WOCN) Society is a continuing education program to improve patient outcomes by enhancing the expertise and support in wound care provided to the hospital. The WTA Program prepares nonspecialty nurses to provide basic, bedside wound care. Three certified WTAs in the NICU played a critical role in the overall HAPI reduction efforts. Based on the success of the early identification of pressure injury risk strategy to emphasize the proactive prevention-focused approach, a plan was set for active surveillance implementation in all ICUs at least once per month and increased to weekly by July 2020. If gaps or deficiencies in care occurred, then reinforcement of best practice skin care bundles were performed in real-time at the bedside.

## DATA COLLECTION

Data were collected on all pressure injuries using the electronic health record (EHR) and internal hospital safety reporting system. In addition to collecting information on all high-stage HAPI, we collected data on all pressure injuries (Stages 1–4 and unstageable) to determine if improved early identification of high-stage pressure injury would unintentionally lead to an increase in lower stage injury (balancing measure). RNs and RTs were trained to assess the skin for redness under and around devices to improve the information submitted to the safety reporting system and patient outcomes. If nonblanchable redness was noted, staff were required to enter a safety report, take a photograph of the affected area, notify their leadership, and request a WOCN consult. Each pressure injury was evaluated and staged by a WOCN. The HAPI occurrence was tracked and calculated as a rate per 1,000 patient days for all patients, including our NICU population, by the QM team. Each improvement team completed checklists for the corresponding continuous EEG and respiratory devices to identify failures or barriers to completion and potential high-risk patients. Nursing members of the quality improvement team performed the audits, and results were reported back to the group. Real-time feedback was given to the bedside nurse when elements of the bundle were missing or not optimal. For respiratory injuries, before the initiation of this project, no preventive data were tracked, only information that indicated a pressure injury had occurred. In addition, mucosal injuries within the nares were not previously included in the data. RTs monitored bundle compliance of patients on CPAP through weekly in-person rounding and monthly chart audits, following up with education to staff who were noncompliant by just-in-time intervention or email. Compliance was reported back to the respiratory therapy device-related pressure injury QI group. The WTA collected active surveillance data at the time of evaluation, and the QM monitored these audits, including reasons for noncompliance.

## MEASURES

The high-stage HAPI rate is the number of stage 3, 4 and unstageable pressure injuries in the numerator per 1,000 patient days in the denominator. EEG-related and CPAP-related high-stage HAPI rates are the number of stage 3, 4 and unstageable pressure injuries that were attributed to those devices per 1,000 patient days. Patient days are counted from the census at midnight.

## ANALYSIS

Measures were analyzed using statistical process control displayed on U-charts. The analysis of these measures adhered to rule-based conventions for special cause variation as defined by Provost and Murray.^21,22^

### Ethical Considerations

Upon discussion with the University of California San Diego Health Human Research Protections Program (HRPP) staff, this quality improvement project was deemed non-human subjects research and therefore exempt from Institutional Review Board (IRB) review.

## RESULTS

**Fig. 3.**
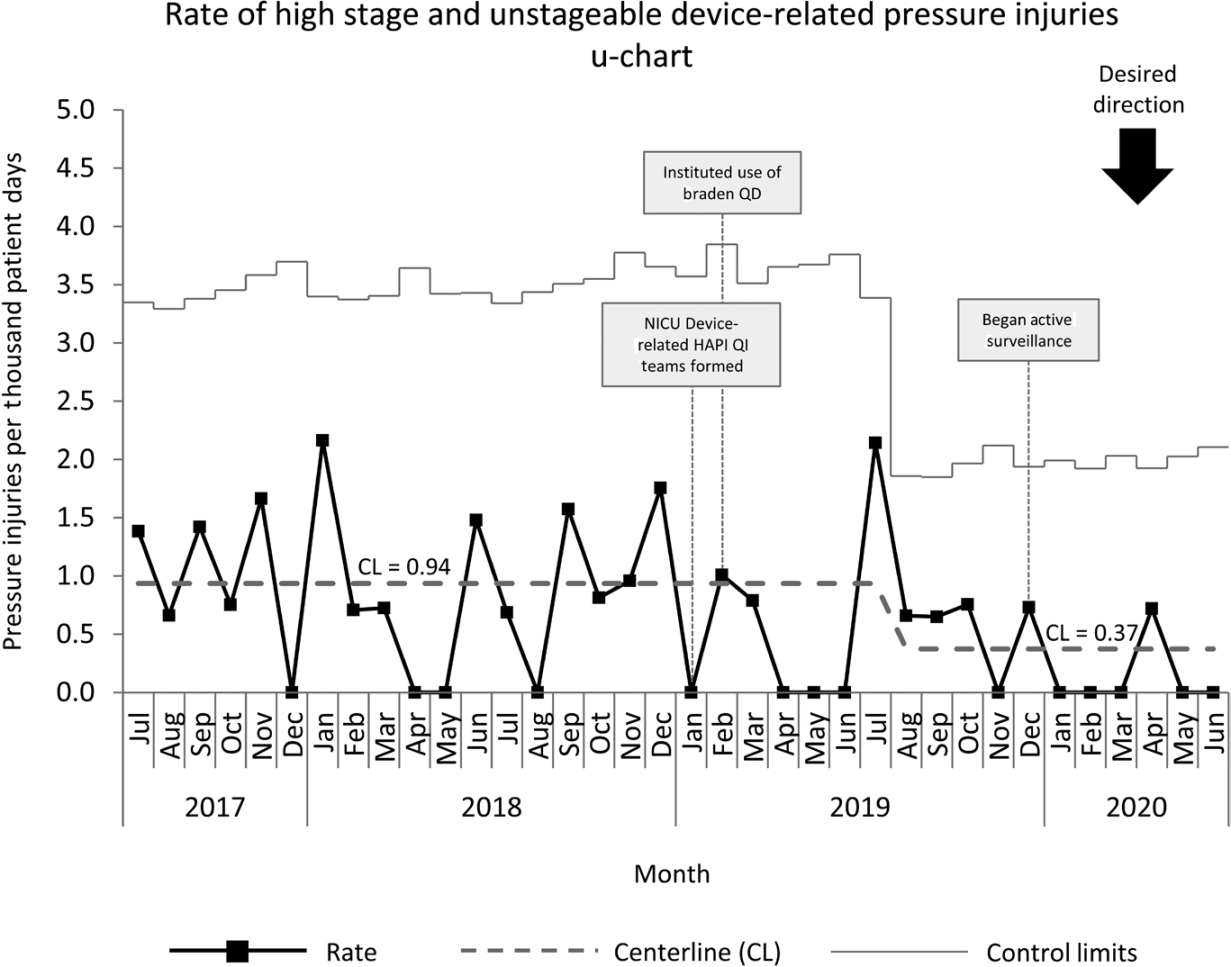
Control chart of high-stage and unstageable device-related HAPI events in the NICU July 2017 to June 2020.

**Fig. 4.**
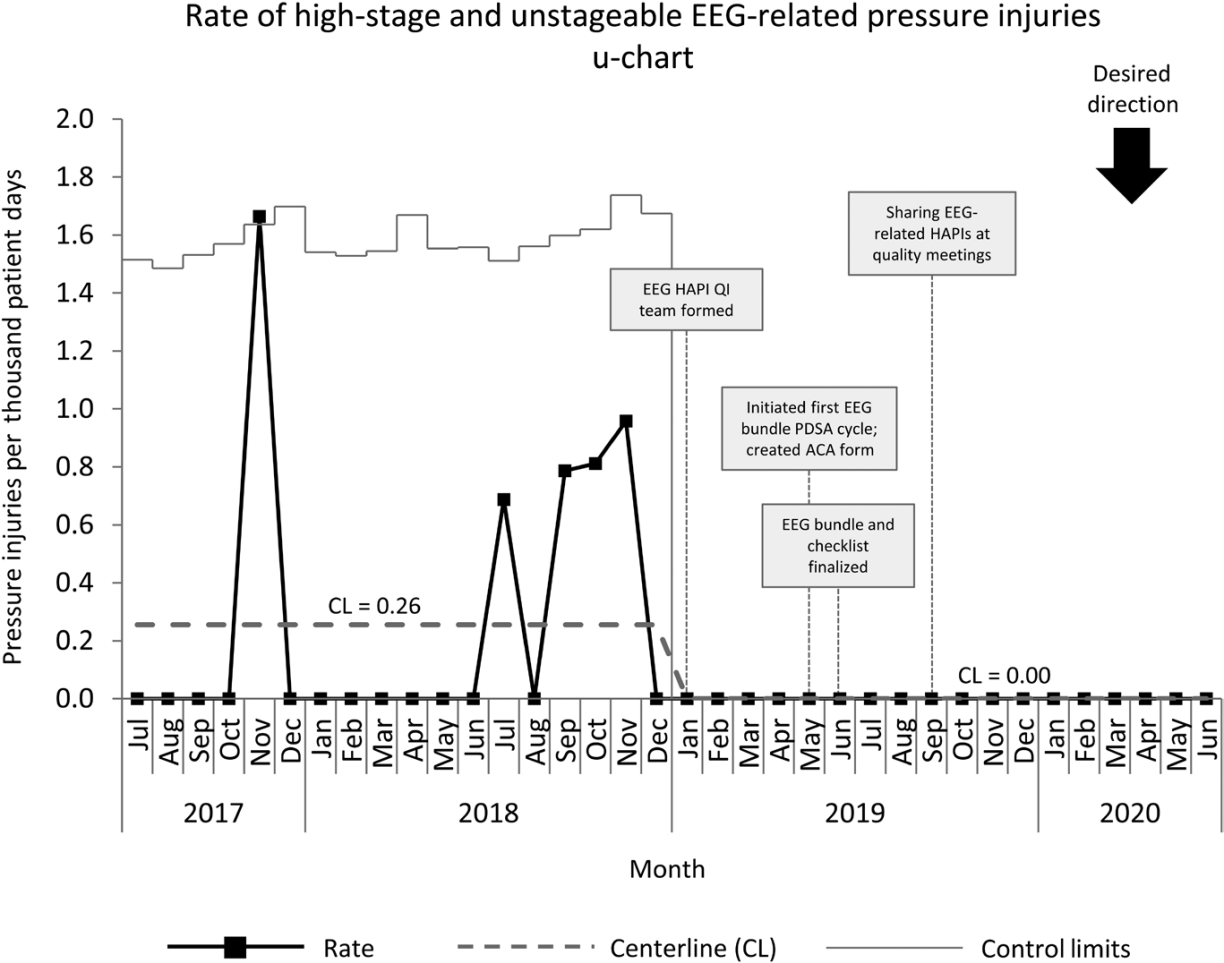
Control chart of high-stage and unstageable EEG-related HAPI events in the NICU July 2017 to June 2020.

**Fig. 5.**
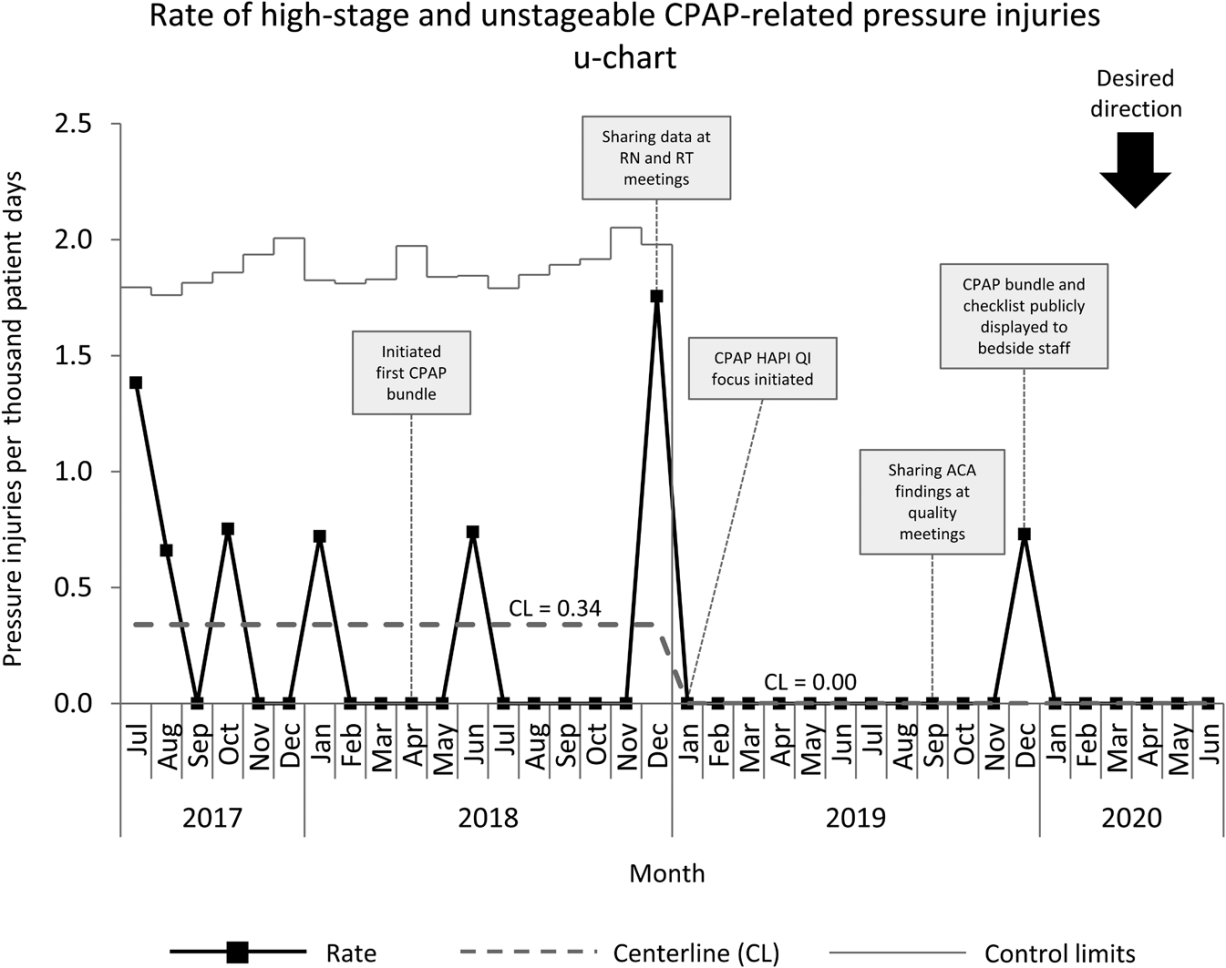
Control chart of high-stage and unstageable CPAP-related HAPI events in the NICU July 2017 to June 2020.

Improvement work in the NICU started in January 2019, with baseline data collected from July 2017 to December 2018. The baseline NICU high-stage HAPI rate of 0.98 per 1,000 patient days was above the SPS collaborative average of 0.107 per 1,000 patient days.^23^ The primary aim of our pressure injury improvement group was to reduce the device-related high-stage HAPI rate per 1,000 patient days by 30% from 0.94 per 1,000 patient days to 0.62 per 1,000 patient days by December 2019. We exceeded this goal by reducing our device-related high-stage HAPI rate by 60% to 0.37 per 1,000 patient days (Fig. 3). EEG-related HAPI and CPAP-related high-stage HAPI rates were reduced to 0 per 1,000 patient days (Figs. 4 and 5). After implementing the EEG bundle, the NICU had no other HAPI events related to EEG devices for over 1 year (Fig. 5).

**Fig. 6.**
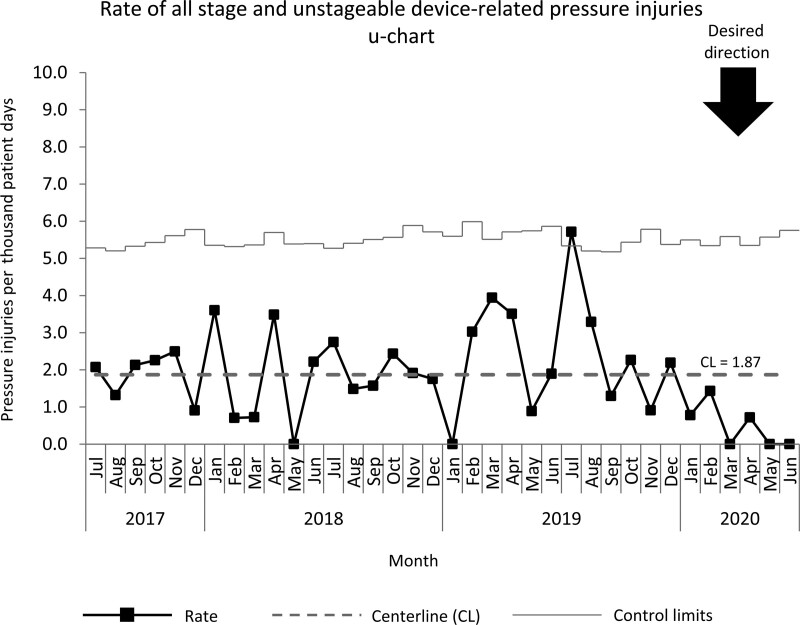
Control chart of all stage device-related HAPI events in the NICU July 2017 to June 2020.

A decrease in all stage HAPIs occurred including those that were device-related, but it was not enough to achieve a centerline shift when normalized to patient days (Fig. 6).

## DISCUSSION

With an emphasis on care standardization, data informed decision-making and interprofessional collaboration, high-stage device-related HAPI’s in the NICU were significantly reduced. Other hospitals have published their success with pressure injury reduction in both the neonatal and pediatric intensive care population,^7,9^ and our focus on device-related pressure injury in the neonatal population expands this body of knowledge. In addition, creating an interprofessional collaboration culture, increasing reliability through cognitive aids such as checklists, and use of timely feedback using ACA forms allowed teams to focus on the highest risk device-related injuries. By having a specific focus, the teams drastically reduced the number of pressure injuries and sustained this reduction using the high-reliability processes that were adopted. Despite 6 months of documented improvement, this interval was insufficient to observe a reduction in the rate of all stage pressure injuries in the NICU. This could be due to a shift from high-stage injuries, where our interventions were focused, to low-stage injuries.^3^

In our hospital, device-related pressure injuries were greater than 80% of all high-stage pressure injuries in our NICU. This is consistent with rates previously reported in the pediatric literature.^2,3,5–7^ By employing a data-driven approach to determine the high-risk areas, our approach targeted interventions with the greatest possibility of reducing HAPIs. Although EEG electrodes and CPAP devices were the highest risk devices for causing HAPI, other devices such as feeding tubes and intravenous tubing also contributed to high-stage HAPIs in our NICU. Using the Pareto chart (Fig. 1) to identify the most frequent kinds of injuries (device-related), we avoided focusing our efforts on the devices infrequently causing events. However, as previously noted, all stage HAPI rates did not significantly decrease which may be related to other types of device-related pressure injury that were unaddressed. This will inspire continued improvement efforts to decrease pressure injury-related harm for all patients.

Pressure injuries related to EEG devices are commonly related to the immobility and acuity of the patient, scalp edema in patients with hypoxic-ischemic encephalopathy, and prolonged, refractory seizures requiring extended EEG monitoring.^6^ Our interventions decreased EEG monitoring time, utilized active surveillance, and decreased pressure on the neonatal scalp significantly reducing high-stage HAPI in our unit.

Early use of Nasal CPAP (NCPAP) following tracheal extubation has been associated with improved neonatal outcomes and decreased bronchopulmonary dysplasia and is considered the standard for respiratory support in preterm infants.^24^ NCPAP is a noninvasive means to provide a constant distending pressure and aids in preventing apnea.^25^ This life-saving technology is not without risks. Pressure injury to the nasal septum is a common complication of NCPAP, occurring in 20%–100% of neonates.^26,27^ NCPAP is a significant risk factor for nasal pressure injury.^26,28–33^ All subjects supported with NCPAP in this project were treated with noninvasive mechanical ventilation (NIMV) or continuous positive airway pressure (CPAP) ventilator modes. Studies on the treatment and prevention of nasal breakdown for infants who require CPAP as respiratory support are minimal, although rotation of the device and use of a pressure barrier are included in the standard prevention bundle elements for SPS.^3,7^ Proper fit and offloading of pressure were recommended elements of the HAPI prevention bundle. The use of a checklist for respiratory devices has been associated with a decrease in pressure injuries rates,^34^ and was utilized in our project.

Active surveillance in the NICU may play a role in preventing high-stage pressure injuries^2,3,7^; by identifying pressure injuries at earlier and lower stages of injury. If pressure injuries are identified sooner, they can be treated and are less likely to develop into a high-stage injury. Active surveillance was clearly one of our most successful interventions to identify high-risk situations, intervene early, and change practices that decreased the risk of pressure injury for each patient.

## LIMITATIONS

This study has several limitations. Although our site is fortunate to have a WTA nurse and WOCN for identifying and staging each pressure injury, these resources are not available at all sites, thus limiting the generalizability of our work to all NICUs. Measures of compliance for each intervention were not completely recorded, and we could not quantify whether one intervention alone was superior to another. Instead, we utilized the quality improvement approach that if an improvement was observed following the introduction of a new intervention, then it was adopted. Multiple PDSA’s occurred simultaneously, and it is difficult to determine which interventions were more effective.

## CONCLUSIONS

Through data-driven decision-making and interprofessional collaboration, our team successfully reduced high-stage device-related pressure injuries in the NICU. This approach can be replicated with expected improvements in other units that experience similar device-related pressure injuries in the NICU.

## DISCLOSURE

The authors have no financial interest to declare in relation to the content of this article.
